# Proteomic Associations and Signatures of Frailty Among Older Men

**DOI:** 10.1093/geroni/igaf122.474

**Published:** 2025-12-31

**Authors:** Jodi Lapidus, Alicia Feryn, Jack Wiendrick, Adam Burns, Eric Orwoll, Kristine Ensrud

**Affiliations:** Oregon Health & Science University, Portland, Oregon, United States; Oregon Health & Science University, Portland, Oregon, United States; Oregon Health & Science University, Portland, Oregon, United States; Oregon Health & Science University, Portland, Oregon, United States; Oregon Health & Science University, Portland, Oregon, United States; University of Minnesota, United States

## Abstract

Aging leads to biological changes that influence frailty onset and prevalence, and these changes are likely reflected in circulating proteins. In this study, we analyzed serum proteomics (SomaLogic 7K panel) from 500 older men (average age 84 ± 3.4 years) in the MrOS study at the fourth visit in 2015. A phenotypic frailty index was computed, including several components: shrinkage (recent weight loss, low BMI), weakness (grip strength <32 kg), low energy, slowness (gait speed <0.8 m/s, use of a walking aid), and low physical activity (no walking for exercise). Participants were classified as robust (22%), intermediate (58%), or frail (20%). Our objective was to identify proteins and co-expression protein modules linked to frailty and create multi-protein signatures of frailty. Seventy-six proteins showed consistent associations with frailty (e.g., odds ratio > |1.5| or standardized effects > 0.3). Frailty-associated proteins included C/EBPβ (linked to inflammation-related diseases), ARIDA1 (involved in DNA repair and stem cell maintenance), APP (related to Alzheimer’s), IL4 (a cytokine regulator in inflammation and wound healing), and AKT1 (a mediator of cellular functions linked to immune issues, proteostasis failure, and cancer). Other frailty-associated proteins were involved in tissue damage/repair (e.g., collagens, TFF3, GDF15) and immune function activation (e.g., CCL15, Cystatin C, B2M). The presentation will also highlight proteins associated with specific frailty components and cluster proteins with similar associations. This research aims to enhance understanding of how proteins and their interconnected biological networks contribute to the multifaceted aspects of frailty in older populations.

